# The Hepatitis C virus NS5A and core proteins exert antagonistic effects on *HAMP* gene expression: the hidden interplay with the MTF‐1/MRE pathway

**DOI:** 10.1002/2211-5463.13048

**Published:** 2020-12-13

**Authors:** Alexios Dimitriadis, Pelagia Foka, Eleni Kyratzopoulou, Eirini Karamichali, Stavroula Petroulia, Panagiota Tsitoura, Athanasios Kakkanas, Petros Eliadis, Urania Georgopoulou, Avgi Mamalaki

**Affiliations:** ^1^ Laboratory of Molecular Biology and Immunobiotechnology Hellenic Pasteur Institute Athens Greece; ^2^ Laboratory of Molecular Virology Hellenic Pasteur Institute Athens Greece; ^3^ Present address: Laboratory of Molecular Biology and Immunobiotechnology Hellenic Pasteur Institute Athens Greece

**Keywords:** HCV, hepcidin, MTF‐1/MRE, NS5A, SMAD4, Zn

## Abstract

Hepcidin, a 25‐amino acid peptide encoded by the *HAMP* gene and produced mainly by hepatocytes and macrophages, is a mediator of innate immunity and the central iron‐regulatory hormone. Circulating hepcidin controls iron efflux by inducing degradation of the cellular iron exporter ferroportin. HCV infection is associated with hepatic iron overload and elevated serum iron, which correlate with poor antiviral responses. The HCV nonstructural NS5A protein is known to function in multiple aspects of the HCV life cycle, probably exerting its activity in concert with cellular factor(s). In this study, we attempted to delineate the effect of HCV NS5A on *HAMP* gene expression. We observed that transient transfection of hepatoma cell lines with HCV NS5A resulted in down‐regulation of *HAMP* promoter activity. A similar effect was evident after transduction of Huh7 cells with a recombinant baculovirus vector expressing NS5A protein. We proceeded to construct an NS5A‐expressing stable cell line, which also exhibited down‐regulation of *HAMP* gene promoter activity and significant reduction of *HAMP* mRNA and hepcidin protein levels. Concurrent expression of HCV core protein, a well‐characterized hepcidin inducer, revealed antagonism between those two proteins for hepcidin regulation. In attempting to identify the pathways involved in NS5A‐driven reduction of hepcidin levels, we ruled out any NS5A‐induced alterations in the expression of the well‐known hepcidin inducers SMAD4 and STAT3. Further analysis linked the abundance of intracellular zinc ions and the deregulation of the MTF‐1/MRE/hepcidin axis with the observed phenomenon. This effect could be associated with distinct phases in HCV life cycle.

## Graphical abstract

During HCV infection, the multifunctional core and NS5A viral proteins regulate the iron‐regulatory hormone hepcidin expression antagonistically. While hepcidin is enhanced by core‐mediated activation of BMP/SMAD and STAT3 pathways, it is down‐regulated by NS5A independently of these factors. Herein, we show that NS5A disturbs the MTF‐1/Zn^2+^/hepcidin positive regulation axis, by mopping up Zn^2+^, thereby preserving the structural integrity of NS5A dimers.

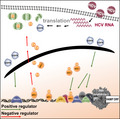

**Abbreviations**
BMP: bone morphogenetic protein
CHC: chronic hepatitis C
Fpn: ferroportin
HAMP: hepcidin antimicrobial peptide
HCC: hepatocellular carcinoma
HCV: hepatitis C virus
Matr2: matriptase‐2
MOI: multiplicity of infection
MRE: metal response element
MTF‐1: MRE‐binding transcription factor‐1
NS5A: nonstructural 5A protein
SMAD4: small mother against decapentaplegic 4
STAT3: signal transducer and activator of transcription 3
TGF: transforming growth factor

The 25‐amino acid long hepcidin is an antimicrobial peptide found in human urine and serum. Its precursor form of 84 amino acids undergoes maturation before it is released in circulation [1]. Hepcidin binds to the only known cell surface iron exporter Fpn expressed in hepatocytes, duodenal enterocytes, and macrophages, triggering the internalization and the consequent degradation of the latter. Thus, hepcidin is able to constrain iron recruitment from the hepatic stores, iron absorption by the duodenum, and macrophage‐dependent iron recycling, and therefore, it is considered as the key peptide hormone that controls iron homeostasis [2, 3].

Hepcidin expression is up‐regulated by iron via the BMP pathway [4, 5] and by infection or inflammation through STAT3 [6]. On the other hand, hepcidin is down‐regulated by elevated erythropoietic activity and low body iron stores [7]. Various other transcription factors engaged in hypoxia, liver‐specific gene transcription, tumorigenesis, and cell differentiation are involved in the regulation of *HAMP* gene expression [8, 9, 10, 11, 12, 13, 14].

Hepatitis C virus belongs to the *Flaviviridae* family together with other highly infectious single‐stranded, positive‐sense RNA viruses. Its 3000‐amino acid polyprotein precursor produces at least ten structural and nonstructural proteins (Core, E1, E2, p7, NS2, NS3, NS4A, NS4B, NS5A, and NS5B), following processing by viral and cellular proteases [15]. HCV infection affects more than 71 million individuals worldwide [16]. 65–80% of them will develop liver inflammation and fibrosis, which are hallmarks of persistent infection. Hepatic steatosis will be detected in more than 40% of CHC patients, who will subsequently deteriorate and develop cirrhosis over a 3‐decade time span. It is estimated that 5% of HCV‐positive cirrhosis patients eventually develop HCC, annually [17].

Deregulation of the iron homeostasis network, encompassing increased serum iron and ferritin levels and elevated intrahepatic iron stores, is often detected in CHC infection [18, 19, 20, 21, 22]. As it turns out, this condition has deleterious effects on the liver, as it causes widespread hepatocyte injury due to induction of oxidative stress, organelle dysfunction, aberrant growth, and onset of liver fibrosis [23].

Hepcidin expression in HCV infection has remained ambiguous so far, with some studies reporting increasing [24, 25] and others decreasing levels [20, 22, 26, 27]. Discrepancies between studies could possibly be attributed to different conditions and subjects of each study. Our previous work revealed a strong positive relationship between viral load and hepcidin serum levels, with acutely infected and chronic patients with high viral load possessing statistically significant elevated hepcidin levels compared with healthy donors. Conversely, chronic HCV patients with low viral load had reduced hepcidin levels. Trying to delineate the molecular mechanisms behind the HCV‐mediated regulation of hepcidin, we demonstrated that HCV nucleoprotein core is responsible for the observed positive modulation of *HAMP* gene expression through the activation of a complex signaling network involving BMP/SMAD and STAT3 pathways and casein kinase II. Furthermore, we showed that this increase of hepcidin in HCV‐infected hepatocytes *in vitro* results in significantly elevated HCV replication [28, 29].

The HCV NS5A is a versatile protein with key roles in viral translation [30], replication [31, 32], and assembly [31, 33]. It consists of three domains, and its multipurposity in HCV virus life cycle is directly linked to its structure; since domains I [34] and II [35, 36] are required for HCV genome replication, domain III has a role in virion assembly [31], whereas all three are involved in HCV RNA translation [37, 38]. Given that NS5A exerts multiple effects on host hepatic gene expression and signaling and that it can either enhance or contradict core protein actions [39] most likely as part of its regulatory activity over particular phases of the viral life cycle, we sought to investigate the effect of this viral protein on hepcidin expression.

## Materials and methods

### Plasmids

The full‐length human −3.1 kb *HAMP* gene promoter was a kind gift from Dr. P. Lee (The Scripps Research Institute, USA) [40]. The expression plasmids pHPI 1430 and pHPI 728 that code for the full‐length core (c191) and NS5A proteins from HCV‐1a strain, respectively, have been described elsewhere [41]. The pCMV5 Flag‐DPC4 (SMAD4) expression plasmid was a gift from Joan Massague (Addgene plasmid # 14039; http://n2t.net/addgene:14039; RRID: Addgene_14039). The pCDNA3.1+/C‐MTF1 and pTK‐Hyg expression plasmids were purchased from GenScript (Piscataway, NJ, USA) and Clontech (Mountain View, California, USA), respectively.

For the construction of the stable Tet‐off NS5A cell line (pTRE‐NS5A), the HindIII fragment from the pHPI 611 plasmid [42] carrying the NS5A sequence‐genotype 1a was cloned into the HindIII site of the pTRE‐tight expression vector.

For the expression of NS5A in HepG2 cells via baculovirus transduction, recombinant baculovirus Bac8119 was generated with the aid of a novel transfer vector (pHPI 8113). Specifically, the new baculovirus transfer vector was initially constructed by ligation of the *Nru*I‐*Eco*RI (1059 bp) fragment from pIREShyg plasmid (Clontech), containing the CMV promoter and a synthetic intron, into the *Stu*I and *Eco*RI sites of the pBacPAK8 vector (Stratagene, La Jolla, California, USA). Subsequently, the *Sph*I‐*Eco*RI fragment from pHPI 691 [43], containing the coding region for HCV NS5A 1a, was cloned into the *Sph*I and *Eco*RI sites of the pHPI 8113 vector, yielding plasmid pHPI 8119. The recombinant baculovirus was generated by cotransfection of Sf9 cells with pHPI 8119 plasmid along with BaculoGold DNA (BD Biosciences) and was further propagated, according to standard protocols [44]. Expression of NS5A in transduced cells was confirmed by western blot analysis, in Huh7 and other cells (data not shown) [41]. The control baculovirus Bac1746 used in this study has been described before [44].

### Cells and transfection assays

The Huh7 and HepG2 hepatoma cell lines were maintained in low glucose DMEM supplemented with 2 mΜ glutamine, 10% (v/v) heat‐inactivated FCS and 100 U·mL^−1^ penicillin/streptomycin. BHK‐21 (Baby Hamster Kidney) cells were maintained in high glucose DMEM supplemented with 2 mΜ glutamine, 10% (v/v) heat‐inactivated FCS, and 100 U·mL^−1^ penicillin/streptomycin. Huh7 cells expressing the tetracycline transactivator (H7TA‐61) (kind gift from Dr. Darius Moradpour) were maintained in high glucose DMEM supplemented with 2 mΜ glutamine, 10% (v/v) heat‐inactivated FCS, 100 U·mL^−1^ penicillin/streptomycin, and 200 µg·mL^−1^ G418.

For the generation of NS5A expressing (NS5A cells) and their relative control (pTRE‐tight) cells, 8000 Η7ΤΑ‐61 cells/well were seeded in 6‐well plates. The cells were simultaneously transfected using JetPEI (Polyplus) with 2 μg pTRE‐NS5A or pTRE‐tight plasmid, and 2 μg pTK‐Hyg to provide hygromycin resistance, according to [45]. After 24 h, the cells were washed with phosphate‐buffered saline and left for 48 h in fresh culture medium. Selection of transfected cells was performed using 200 µg·mL^−1^ G418, 100 µg·mL^−1^ Hygromycin, and 100 µg·mL^−1^ doxycycline. The cells were maintained in medium containing 200 µg·mL^−1^ G418, 40 µg·mL^−1^ Hygromycin, and 100 µg·mL^−1^ doxycycline. Moreover, the control cell line pTRE‐tight was subjected to the same treatment without doxycycline and used for comparison purposes instead of doxycycline‐treated NS5A cells, due to the slight leakiness of protein expression, often observed with the Tet‐Off system. HCV NS5A expression following induction of the NS5A cell clone by removal of doxycycline was examined by immunofluorescence.

For transient transfections, 100 000 cells per well were seeded in 48‐well plates 24 h prior to the experiment. The cells were transfected using JetPEI (Polyplus) with 0.25 μg promoter‐luciferase DNA constructs and the appropriate amount of expression plasmid/empty vector up to 0.1 µg of total DNA, as well as 0.05 μg of CMV‐β‐galactosidase expression plasmid to provide an internal control for transfection efficiency. After 6 h, the cells were washed with phosphate‐buffered saline and left for 48 h in fresh culture medium. Cell lysates were subjected to luciferase and β‐galactosidase activity determination with commercially available kits (Promega, Madison, Wisconsin, USA). Luciferase activity was normalized to β‐galactosidase activity in order to yield relative luciferase activity (RLA). In all figures, the RLA vector control value (mean ± SD: standard deviation) was set as 100% (black bars) and all other values were depicted as a ratio of this.

For baculovirus transduction of HepG2 cells, cells at a density of 5 × 10^5^ were infected with an MOI of 25 for 3 h at room temperature. Following infection, 5 mΜ of sodium butyrate was added in the medium for 24 h. After 48 h of infection, cells were lysed as described previously.

### mRNA expression analysis

Total RNA was isolated from cells using RNAzol B (Wak‐Chemie Medical, Steinbach, Germany), according to the manufacturer’s instructions with the following modification; 1 μg·μL^−1^ glycogen was used to enhance RNA precipitation from isopropanol solutions, before the final wash step in 75% (v/v) ethanol. Reverse transcription reactions were carried out using 1 µg RNA and MMLV reverse transcriptase (Promega). The prepared cDNA was subjected to qPCR using the Kapa^®^ SYBR Fast Master Mix (Kapa Biosystems, Wilmington, Massachusetts, USA) in a Mini Opticon PCR thermocycler (Bio‐Rad, Hercules, California, USA). The gene‐specific primers used were HAMPF: 5′‐CCA CAA CAG ACG GGA CAA CTT‐3′, HAMPR: 5′‐AGT GGG TGT CTC GCC TCC TT‐3′. Primers for 18S rRNA have been described elsewhere [46]. Results were analyzed with the internal standard‐curve method and normalized to 18S rRNA to provide the relative mRNA expression. In all PCR experiments, the relative mRNA expression control value (mean ± SD) was set as 100% (black bar) and all other values were depicted as a ratio of this.

### Protein and immunofluorescence analysis

Cells were washed twice with phosphate‐buffered saline and harvested in whole‐cell extract lysis buffer (10 mm Tris/HCl pH 7.05, 50 mm NaCl, 1% (w/v) Triton X‐100, 0.5 mΜ PMSF and protease/phospho‐protease inhibitor cocktails by Roche). Protein concentrations were measured with the MicroBCA assay (Thermo Scientific). 40 μg of cell lysates were resolved in 10% (v/v) SDS/PAGE gels and transferred onto nitrocellulose membranes. After blocking, membranes were incubated overnight with primary antibodies. Membranes were then washed and incubated with the appropriate secondary antibody for 90 min at room temperature. Chemiluminescence was detected using Pierce ECL western blotting substrate (Thermo Scientific).

The following antibodies were used in this study: SMAD4 (#9515) by Cell Signaling (Danvers, Massachusetts, USA); TMPRSS6 (ab56180), by Abcam (Cambridge, UK); β‐actin (MAB1501) by Millipore (Burlington, Massachusetts, USA); ferroportin 1 (MTP11) by Alpha Diagnostics (San Antonio, Texas, USA); and STAT3 (sc‐483) and HepC Cag (C7‐50) by Santa Cruz Biotechnology (Dallas, Texas, USA). The polyclonal HCV NS5A and HCV‐1a core antibodies used in this study have been described before [43, 47].

Hepatitis C virus NS5A expression following induction of the cell clones was examined by immunofluorescence with the HCV NS5A polyclonal antibody, as previously described [41].

### Enzyme‐Linked Immunosorbent Assays (ELISA)

Quantification of secreted hepcidin levels was performed by home‐made competitive ELISA, as earlier reported [48]. Protein expression of the control cells was set as 100% (black bars), and all other values were calculated as a percentage of the control.

### Statistical analysis

Statistical analysis was performed using Student’s *t*‐test with *P* ≤ 0.05 considered as statistically significant (*P‐value ≤ 0.05; **P‐value ≤ 0.005). Unless otherwise shown, statistical analysis was carried out between control and treated cells.

## Results

### HCV NS5A protein down‐regulates hepcidin expression

**Fig. 1:**
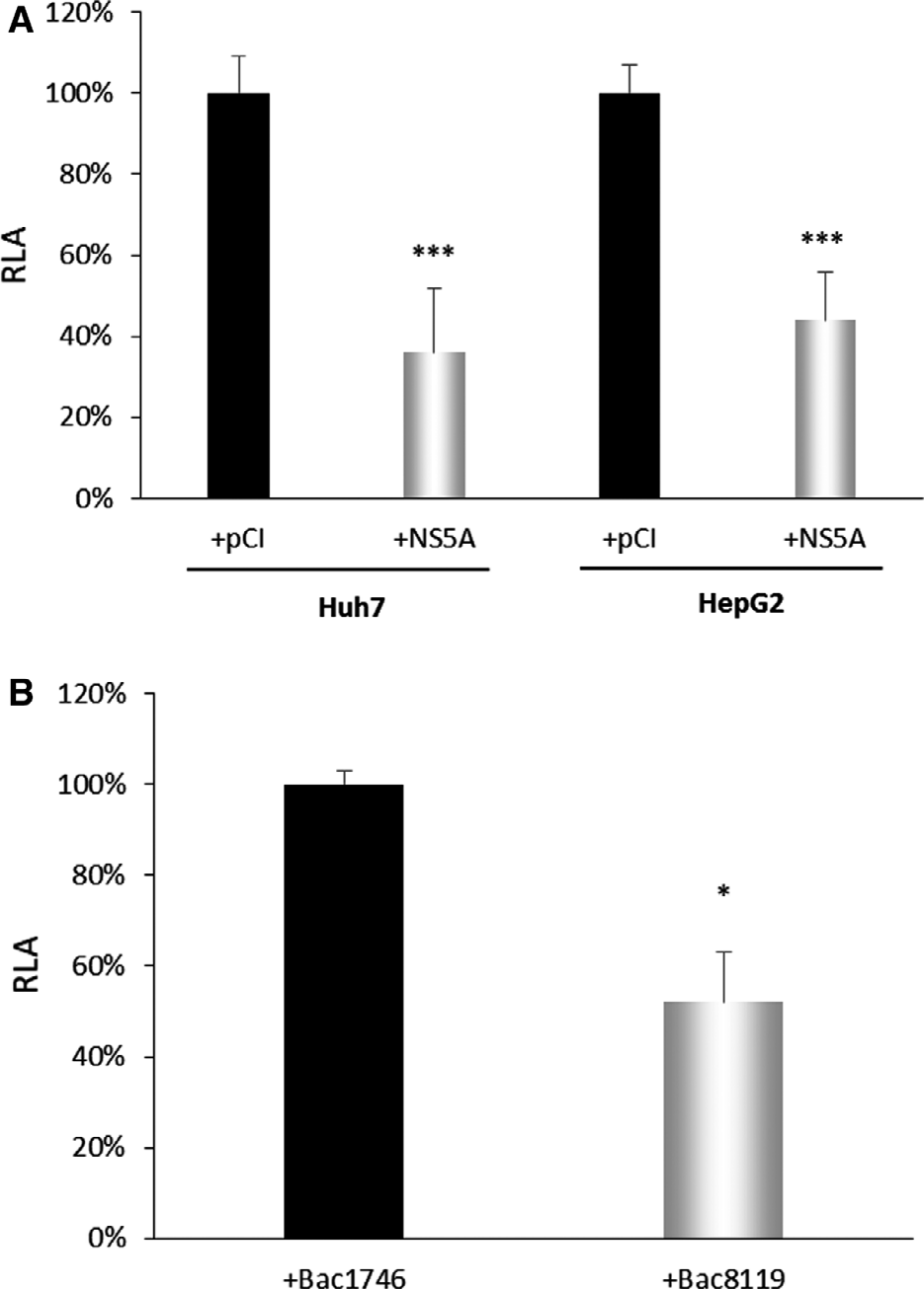
HCV NS5A protein decreases the activity of *HAMP* gene promoter. (A) Transient cotransfections of Huh7 and HepG2 cells with the −3.1 kb *HAMP* gene promoter reporter construct and the expression plasmids encoding either full‐length NS5A or the empty vector (pCI) (black bar). The normalized luciferase activity (RLA) 48 h post‐transfection of the vector has arbitrarily been assigned as 100% (black bar), with that of the HCV NS5A protein being represented with respect to this value. (B) Huh7 cells transiently transfected with the full‐length *HAMP* construct were transduced with baculovirus Bac8119 expressing the NS5A protein or the control baculovirus Bac1746. The normalized luciferase activity (RLA) 72 h post‐transduction of cells transduced with Bac1746 has arbitrarily been assigned as 100% (black bar), with that of the HCV NS5A protein being represented with respect to this value. Error bars denote mean ± SD and significance was calculated by Student’s *t*‐test, with *P*‐value ≤ 0.05 considered as statistically significant (*P‐value ≤ 0.05; **P‐value ≤ 0.005). Each experiment was carried out at least 3 times in triplicate.

The effect of HCV NS5A protein on *HAMP* gene expression was evaluated with its ectopic expression in hepatoma cells by transient transfection, and the subsequent assessment of the relative *HAMP* gene promoter activity. To elaborate, Huh7 and HepG2 cells were cotransfected with the full‐length −3.1 kb *HAMP* gene promoter and pHPI 728 NS5A‐coding expression plasmid. Figure 1A shows that in both types of cells, HCV NS5A decreased the activity of *HAMP* promoter by 60%, as compared to the empty vector control. To increase cell transfectability, Huh7 cells transiently transfected with the above mentioned *HAMP* gene promoter construct were transduced with BAC8119. This experiment further verified the HCV NS5A‐mediated down‐regulation of *HAMP* gene promoter (Fig. 1B). Figure S1A,B depicts HCV NS5A protein levels in transfected and virally transduced cells, respectively.

**Fig. 2:**
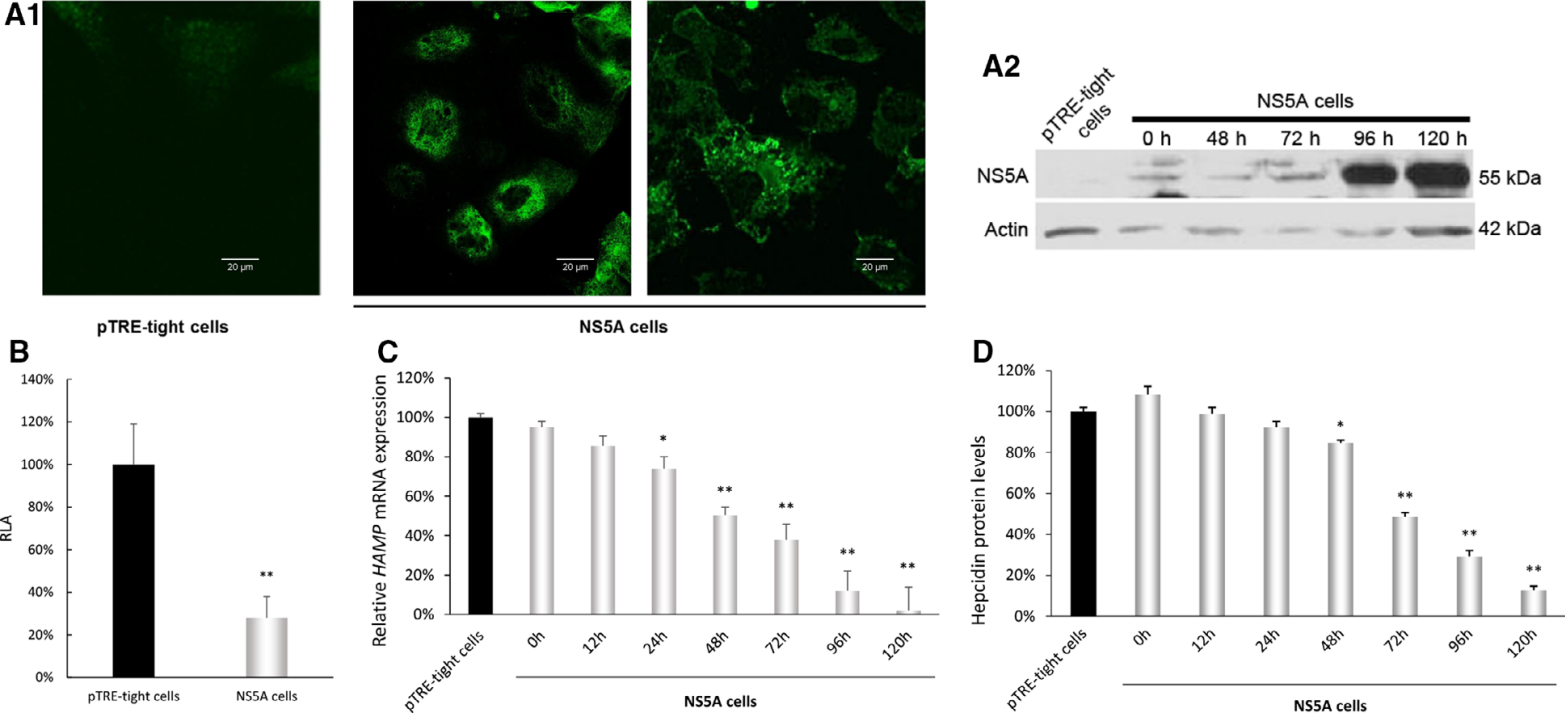
HCV NS5A down‐regulates *HAMP* gene expression. (A) An inducible Huh7 Tet‐off cell line expressing the full‐length NS5A protein upon withdrawal of the antibiotic selection marker (NS5A cells) was constructed, and the expression of NS5A was monitored with confocal microscopy at 96 h (A1) and time‐course of NS5A protein expression analyzed in whole‐cell extracts by western blotting analysis (A2) with antibody against NS5A. pTRE cells: cells stably transfected with the empty pTRE‐tight vector. The two images of NS5A cells in A1 represent different field of the same slide. The bar length is 20 μm. Actin in A2 was used as an internal control. Polypeptide molecular mass are given on the side in kDa. (B) pTRE‐tight and NS5A cells were transiently transfected with the −3.1 kb *HAMP* gene promoter reporter construct. The normalized luciferase activity (RLA) 48 h post‐transfection of pTRE‐tight cell extracts has arbitrarily been assigned as 100% (black bar) with that of the HCV NS5A protein being represented with respect to this value. Error bars denote mean ± SD and significance was calculated by Student’s *t*‐test, with *P*‐value ≤ 0.05 considered as statistically significant (*P‐value ≤ 0.05; **P‐value ≤ 0.005). The experiment was carried out 3 times in triplicate. (C) *HAMP* mRNA levels during an HCV NS5A expression time‐course experiment. Total RNA was isolated at various time points following induction of NS5A expression and subjected to RT‐qPCR with *HAMP* gene‐specific primers. The histogram depicts *HAMP* relative mRNA expression during the course of NS5A induction for 120 h. pTRE‐tight mRNA expression at 120 h was arbitrarily set as 100% (black bar) with all other values being represented as a ratio of this. Error bars denote mean ± SD and significance was calculated by Student’s *t*‐test, with *P*‐value ≤ 0.05 considered as statistically significant (*P‐value ≤ 0.05; **P‐value ≤ 0.005). The experiment was carried out 3 times in triplicate. (D) Secreted hepcidin protein levels from pTRE‐tight and NS5A cell supernatants measured by a competitive ELISA assay during the course of 120 h. The value of pTRE‐tight protein expression at 120 h was arbitrarily set as 100% (black bar) with all other values being represented as a ratio of this. Error bars denote mean ± SD and significance was calculated by Student’s *t*‐test, with *P*‐value ≤ 0.05 considered as statistically significant (*P‐value ≤ 0.05; **P‐value ≤ 0.005). The assay was repeated three times in quadruplicates.

In order to verify the effect of HCV NS5A protein on *HAMP* gene expression, we used the inducible Huh7 Tet‐off cell line expressing the full‐length NS5A protein upon withdrawal of the antibiotic selection marker doxycycline for up to 120 h. Induction of NS5A was confirmed by immunofluorescence (Fig. 2A1) and western blot assay (Fig. 2A2). When the NS5A expressing cells were transfected with the −3.1 kb *HAMP* gene promoter, the activity was once again reduced, in contrast to the pTRE‐tight control cells (Fig. 2B).

Subsequently, we sought to verify the effect of HCV NS5A on endogenous *HAMP* mRNA levels by RT‐qPCR in NS5A‐expressing cells and its pTRE‐tight control cells. Figure 2C reveals that *HAMP* mRNA decreased gradually over time, reaching only about 10% or less of the control levels and in line with the maximal increase of NS5A, 120 h post‐induction. Hepcidin secreted peptide levels were also determined by ELISA in the supernatants of NS5A‐overexpressing and control cells and showed a similar drop, suggesting a clear HCV NS5A‐mediated transcriptional regulation of *HAMP* gene expression (Fig. 2D). The observed hepcidin concentration was found to be 27.58 ± 0.57 ng·mL^−1^ for pTRE‐tight cells and dropped to 3.41 ± 0.08 ng·mL^−1^ in NS5A cell supernatants. Experiments carried out in the stable cell lines in the presence of doxycycline failed to procure a reduction in hepcidin expression (data not shown). Collectively, the above presented data clearly demonstrate an HCV NS5A‐mediated transcriptional regulation of *HAMP* gene expression.

**Fig. 3:**
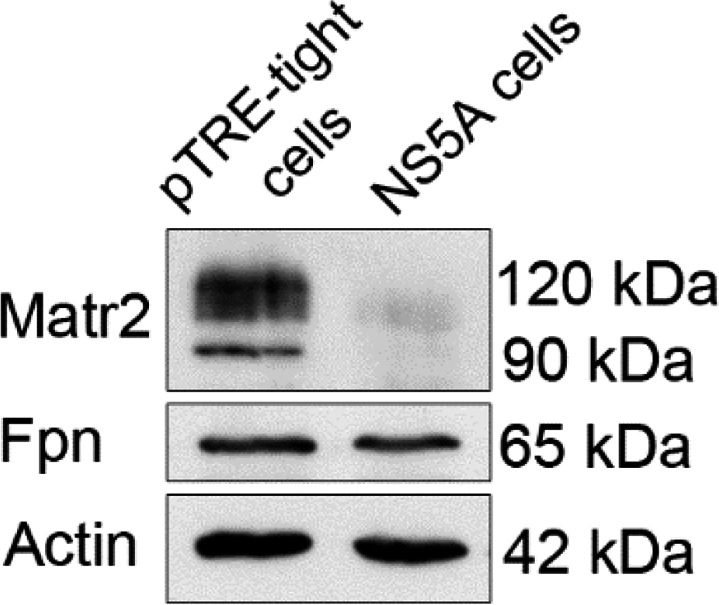
HCV NS5A protein affects components of iron homeostasis network. Western blot analysis of whole‐cell extracts from pTRE‐tight and NS5A cells with antibodies against the iron‐regulatory pathway proteins Fpn and Matr2. Actin was used as an internal control. Polypeptide molecular weights are given on the side in kDa. Individual gel photographs presented in this figure panel depict results from samples that were derived from the same experiment and processed in parallel. Additionally, the loading control was run on the same blot as the primary antibodies.

Interestingly, western blot analysis in whole‐cell extracts from NS5A‐expressing cells and their controls revealed that the main cellular target of hepcidin, the iron exporter Fpn remained intact, while the negative hepcidin regulator Matr2 was dramatically down‐regulated (Fig. 3). These results are consistent with the role of these proteins in iron homeostasis network and the observed decrease in hepcidin transcription and secretion.

### HCV NS5A attenuates the HCV core‐mediated increase of *HAMP* gene expression

**Fig. 4:**
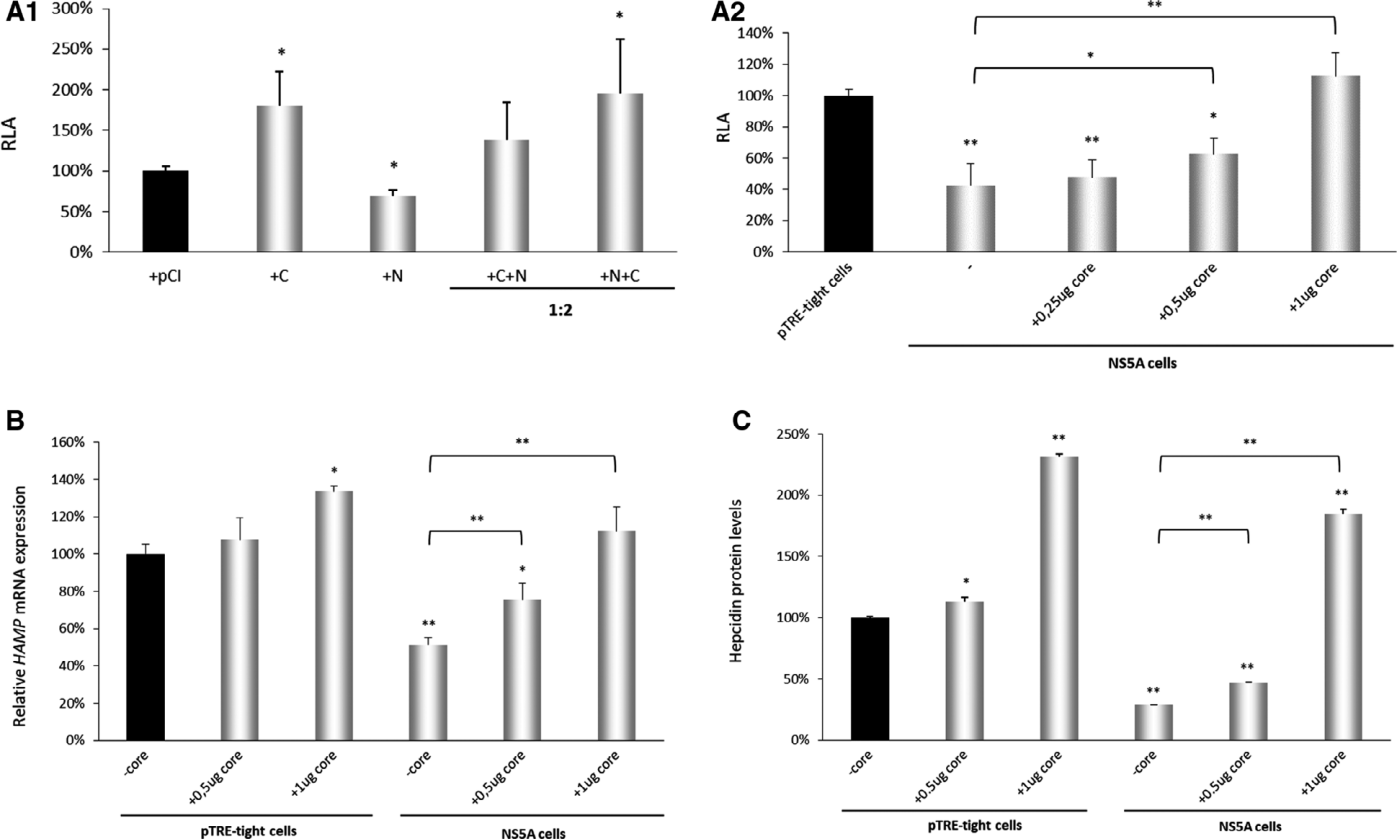
HCV NS5A and core proteins exert an antagonistic effect on *HAMP* gene expression. (A1) Huh7 cells were transiently cotransfected with the −3.1 kb *HAMP* gene promoter reporter construct, and expression plasmids coding for the NS5A protein (N), the core protein (C) or the empty pCI vector, either individually, or in combination at the stated ratios. The normalized luciferase activity (RLA) 48 h post‐transfection of the vector has arbitrarily been assigned as 100% (black bar), with that of the other transfected proteins being represented with respect to this value. (A2) NS5A and pTRE‐tight cells were transiently cotransfected with the −3.1 kb *HAMP* gene promoter reporter construct and different amounts of the expression plasmid coding for the full‐length HCV core protein. The normalized luciferase activity (RLA) 48 h post‐transfection of the pTRE‐tight cells has arbitrarily been assigned as 100% (black bar), with all other values being represented with respect to this value. Error bars denote mean ± SD and significance was calculated by Student’s *t*‐test, with *P*‐value ≤ 0.05 considered as statistically significant (*P‐value ≤ 0.05; **P‐value ≤ 0.005). Each experiment was carried out at least 3 times in triplicate. (B) *HAMP* mRNA levels in pTRE‐tight and NS5A cells transiently transfected with different amounts of the expression plasmid coding for the full‐length HCV core protein. Total RNA was isolated 48 h post‐transfection and subjected to RT‐qPCR with *HAMP* gene‐specific primers. *HAMP* mRNA expression in pTRE‐tight cells was arbitrarily set as 100% (black bar) with all other values being represented as a ratio of this. Error bars denote mean ± SD and significance was calculated by Student’s *t*‐test, with *P*‐value ≤ 0.05 considered as statistically significant (*P‐value ≤ 0.05; **P‐value ≤ 0.005). The experiment was carried out 3 times in triplicate. (C) Secreted hepcidin protein levels in supernatants from pTRE‐tight and NS5A cells transiently transfected with different amounts of the expression plasmid coding for the full‐length HCV core protein, measured by a competitive ELISA assay 48 h post‐transfection. The value of pTRE‐tight protein expression was arbitrarily set as 100% (black bar) with all other values being represented as a ratio of this. Error bars denote mean ± SD and significance was calculated by Student’s *t*‐test, with *P*‐value ≤ 0.05 considered as statistically significant (*P‐value ≤ 0.05; **P‐value ≤ 0.005). The assay was repeated three times in quadruplicates.

Our previous studies revealed that HCV core protein, standing at the forefront of virus–host interactions, positively regulates hepcidin expression and affects components of the iron homeostasis network [29]. In the light of these results, it was intriguing to investigate whether HCV NS5A and core proteins would exert an antagonistic effect on *HAMP* gene expression. Therefore, we cotransfected Huh7 hepatoma cells with the full‐length −3.1 kb *HAMP* gene promoter reporter construct together with the pHPI 1430 plasmid expressing the HCV core protein and the pHPI 728 plasmid expressing the NS5A protein at different mass ratios and measured promoter activity by luciferase assay. Figure 4A1 reveals that while the HCV core protein increased *HAMP* gene promoter activity almost twofold, as seen before [29], the concurrent expression of core and NS5A eradicates the observed effects when NS5A is in excess. Subsequently, the *HAMP* gene promoter construct was cotransfected together with different amounts of the pHPI 1430 plasmid coding for HCV core, in the NS5A‐expressing cell line. Again, increasing amounts of core protein were able to invert the NS5A‐mediated down‐regulation of *HAMP* gene promoter and even abrogate it at a threshold of 1 µg of HCV core expression plasmid (Fig. 4A2). Measurements of hepcidin mRNA (Fig. 4B) and peptide levels (Fig. 4C) using total RNA and supernatants from the same experiments corroborated the observed effect. At the same time, results from the cotransfection of the pTRE‐tight control cells were in line with the already published data concerning the effect of HCV core protein on the *HAMP* gene expression [29]. Figures S1B1,B2 demonstrate the different expression levels of HCV core and HCV NS5A proteins upon transient transfection of the corresponding expression plasmids at the described mass ratios. Taken together, the above results demonstrate that the antagonistic effect of HCV NS5A and core protein on *HAMP* gene promoter activity hints toward the necessity for fine tuning of the iron homeostasis network through tight regulation of hepcidin expression during HCV infection.

### Hepcidin decrease is independent of NS5A‐mediated suppression of SMAD4 protein expression

**Fig. 5:**
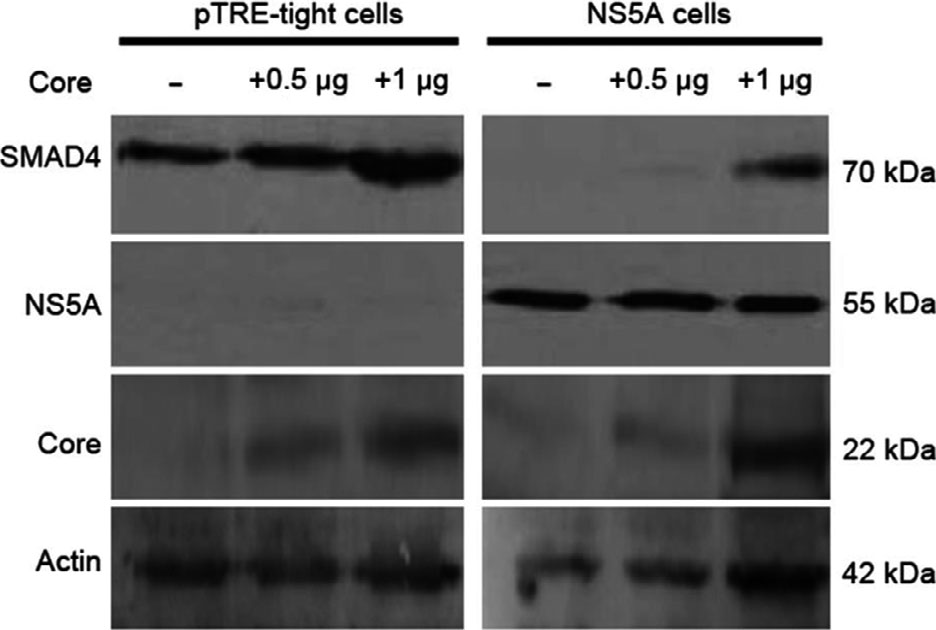
HCV NS5A protein suppresses SMAD4 protein expression. Western blot analysis of whole‐cell extracts from pTRE‐tight and NS5A cells transiently transfected with different amounts of the expression plasmid coding for the full‐length HCV core protein, against the constitutively phosphorylated SMAD4 protein. The expression of NS5A protein, core protein, and actin was monitored as internal control. Polypeptide molecular weights are given on the side in kDa. Individual gel photographs presented in this figure panel depict results from samples that were derived from the same experiment and processed in parallel.

Previous studies from our laboratory have shown that HCV core up‐regulates *HAMP* gene expression through SMAD4 and STAT3 signaling [29]. Given the well‐documented HCV NS5A‐mediated modulation of the STAT3 pathway [49, 50, 51], which we replicated in our own NS5A‐expressing cell line (Figure S1C), we examined whether SMAD4 was implicated in the NS5A‐induced hepcidin down‐regulation. Western blot analysis using pTRE‐tight and NS5A whole‐cell extracts revealed that HCV NS5A protein led to a dramatic decrease of SMAD4 expression levels, 72 h post‐ induction when NS5A protein is overexpressed. As expected, ectopic expression of increasing amounts of HCV core could partially overcome the nullifying effect of NS5A on these proteins (Fig. 5). Unfortunately, transfection of a SMAD4 overexpression plasmid in these stable cell lines was not able to abrogate the NS5A‐mediated down‐regulation of *HAMP* gene promoter activity (data not shown). Thus, it is possible that although NS5A protein suppresses SMAD4 signaling, there are other factors involved in the observed NS5A‐induced alteration of hepcidin gene expression.

### Implication of the MTF‐1/MRE signaling pathway in the NS5A‐mediated down‐regulation of *HAMP* gene expression

**Fig. 6:**
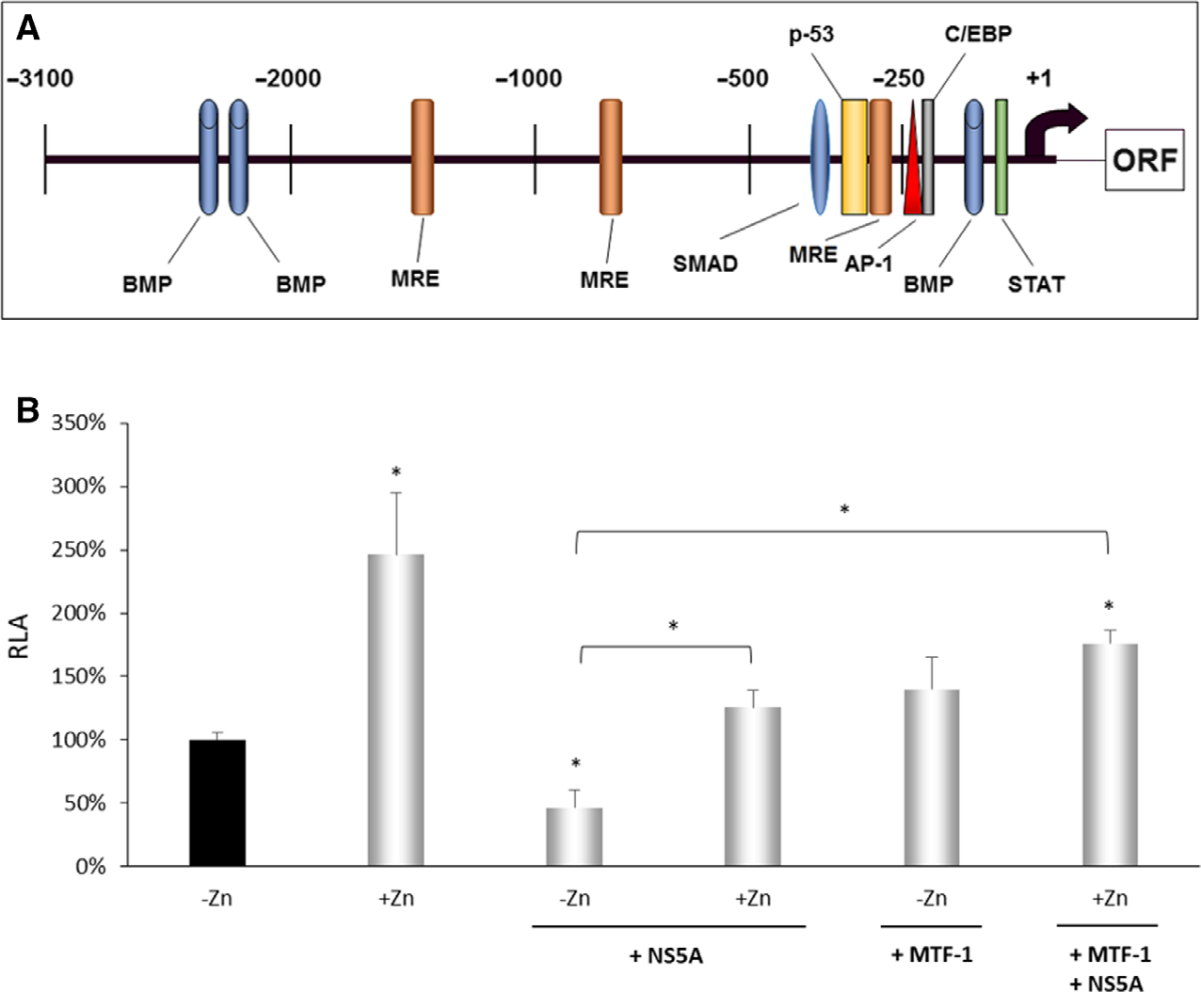
HCV NS5A protein down‐regulates *HAMP* gene expression through the MTF‐1/MRE transcriptional axis. (A) Schematic diagram of the *HAMP* gene promoter region with transcription factor binding sites. The black arrow denotes the transcription initiation site. ORF: hepcidin open reading frame. (B) BHK‐21 cells were transiently cotransfected with the −3.1 kb *HAMP* gene promoter reporter construct, and expression plasmids coding for the NS5A protein and/or the MTF‐1 transcription factor in the presence/absence of ZnCl_2_ (Zn). The RLA value 48 h post‐transfection from zinc‐untreated cells has been arbitrarily assigned as 100% (black bar), with all other values being represented with respect to this value. Error bars denote mean ± SD and significance was calculated by Student’s *t*‐test, with *P*‐value ≤ 0.05 considered as statistically significant (*P‐value ≤ 0.05; **P‐value ≤ 0.005). The experiment was carried out 3 times in triplicate.

It is well documented that HCV NS5A is a zinc metalloprotein [52]. Since Balesaria *et al*. reported that divalent metal ions, like zinc, regulate hepcidin gene transcription via interactions between functional MREs in the hepcidin promoter (Fig. 6A) and the MTF‐1 transcription factor [53], one explanation for NS5A‐induced *HAMP* gene down‐regulation could be that the viral protein may function as a ‘zinc ion sponge’, thereby reducing hepcidin gene transcription. To test this hypothesis, we incubated NS5A‐expressing cells and their controls with ZnCI_2_ at a final concentration of 100 μm and 6 h later the cells were harvested. RT‐qPCR analysis revealed that hepcidin mRNA levels remained low in NS5A cells even after the addition of ZnCl_2_ (data not shown). Similarly, the presence of ZnCl_2_ could not abrogate the NS5A‐mediated effect on *HAMP* gene promoter in cotransfection experiments with the NS5A expression plasmid (data not shown).

Due to the main role of the liver in heavy metal detoxification processes [54, 55], we decided to repeat the aforementioned experiments with nonhepatic cell lines, such as the BHK‐21 cells. Addition of ZnCl_2_ in the medium of control BHK‐21 cells led to a 2.5‐fold up‐regulation of *HAMP* gene promoter activity, indicating the suitability of this cellular system for zinc studies (Fig. 6B). Overexpression of HCV NS5A led to a significant decrease in luciferase activity, as expected, while supplementation with Zn^2+^ ions inhibited the NS5A‐exerted effect. Furthermore, cotransfection of MTF‐1 with NS5A expression plasmids not only abrogated the NS5A‐induced down‐regulation of *HAMP* gene promoter in the presence of zinc ions, but also increased promoter activity by approximately twofold, thus confirming the implication of the MTF‐1/MRE transcriptional axis in the observed effect.

## Discussion

NS5A has the ability to interact with more than 130 host‐cell proteins under certain conditions and in response to different stimuli [31], thus controlling the host‐cell pathways and facilitating virus propagation. For that reason, one may assume that NS5A could manipulate the iron homeostasis network, which is vital for HCV proliferation [28]. Indeed, in our hands NS5A protein was able to transcriptionally diminish the expression of the iron‐regulatory protein hepcidin, since the effect was prominent on *HAMP* gene promoter as well as on the hepcidin mRNA and peptide levels. Consistently, levels of the main cellular target of hepcidin, ferroportin, were not altered. At the same time, the negative hepcidin regulator matr2 was dramatically down‐regulated, a seemingly peculiar observation. However, Belot and colleagues have reported that the endoplasmic reticulum (ER) stress suppresses Matr2 expression at the transcriptional level [56]. Given that the accumulation of HCV NS5A into the cell triggers the endoplasmic reticulum stress [49], the detected down‐regulation of Matr2 could be attributed to the NS5A‐induced ER stress and is not directly linked to altered iron levels [57].

Previous studies from our laboratory concerning the action of HCV core protein on the iron homeostasis network implicated the two major signaling pathways BMP/SMAD and STAT3 in the core‐driven hepcidin regulation. In those experiments, expression of total and phosphorylated STAT3 in Tyr^705^ and Ser^727^ was found up‐regulated by HCV core [29]. STAT3 has also been shown to be activated by phosphorylation in both Ser and Tyr residues in HCV NS5A‐transiently transfected hepatoma cells, as well as in the liver of NS5A transgenic mice [49, 50, 51]. As we did not observe any changes in STAT3 levels in our NS5A‐expressing cells either, we ruled out a putative involvement of this transcription factor in the NS5A‐mediated decrease of *HAMP* gene expression. At the same time, another member of the JAK/STAT family, STAT1, has been demonstrated to be involved in IFN‐γ‐inducible regulation of *HAMP* gene expression in macrophages and other cells [58, 59]. However, given that STAT1 activation is known to be inhibited by both HCV core and NS5A via blockage of its import into the cell nucleus [60] and that we observed a differential modulation of these viral proteins on hepcidin expression, we hypothesized that STAT1 might not be involved in the HCV NS5A‐mediated down‐regulation of *HAMP* gene expression. Therefore, we concentrated our efforts on the investigation of the BMP/SMAD pathway involvement in our effect and, surprisingly, we detected a potent NS5A‐induced down‐regulation in the expression of SMAD4, a finding that has never been reported before and could result in TGF‐β signaling inhibition. Choi and Hwang reported that NS5A was able to inhibit the TGF‐β signaling cascade via interaction with TGF‐β receptor I (TβR‐I) and blockade of multiple SMAD‐related steps of the pathway. However, analysis of the endogenous SMAD4 expression was not conducted in that study [61]. Transfection of NS5A‐expressing cells with a SMAD4 expression plasmid had no success in overturning hepcidin reduction, thereby indicating that the BMP/SMAD pathway is not involved in the NS5A‐mediated modulation of hepcidin gene expression.

Interestingly, the analysis of domain I of NS5A revealed the existence of a tetracysteine motif capable of coordinating the binding of a zinc atom in the N terminus of the protein, making NS5A a metalloprotein [52]. This motif is vital for NS5A function, since its mutation led to the abolishment of the zinc interaction and the consequent abrogation of HCV genome replication. Hepcidin, on the other hand, seems to be a pleiotropic sensor of divalent metals, zinc included, as shown by Balesaria and colleagues [53]. Their study demonstrated that in response to zinc treatment, MTF‐1 transcription factor binds to three consensus MRE *cis*‐acting elements in the −1.8 kb *HAMP* gene promoter, with the first one consisting of two overlapping elements in reverse orientation, thereby modulating hepcidin promoter activity. Therefore, we hypothesized that the expression of the viral protein leads to divalent metal ion binding on its zinc‐binding motif, triggering the reduction of metal ions from the cells, and the subsequent dissociation of MTF‐1 from MRE elements on hepcidin promoter. Nevertheless, it was not possible for us to test whether supplementation of ZnCl_2_ in the medium of NS5A‐expressing hepatoma cells could reverse the observed reduction in hepcidin mRNA. It is well known that the hepatic cell lines constitutively express metallothionein‐1 (MT‐1) [54, 62], a protein that plays a key role in the protection against metal toxicity and oxidative stress, through its ability to bind both physiological and xenobiotic heavy metals and ions [55, 63]. The utilization of a cell line like BHK‐21, which has decreased endogenous MT‐1 expression and, as a result, exhibits a lower capacity to buffer zinc intracellularly [53], was key in demonstrating that exogenously added zinc could abrogate the NS5A‐induced reduction in *HAMP* gene promoter activity. Moreover, the concurrent overexpression of the transcription factor MTF‐1 led to a complete inversion of the effect, supporting the notion that the NS5A‐triggered down‐regulation of *HAMP* gene expression could be attributed to the depletion of Zn^2+^ ions and the subsequent inactivation of the MTF‐1/MRE axis.

The relationship between HCV core and NS5A has been extensively studied in the past. It has been reported that these two viral proteins colocalize on the surface of lipid droplets [64] and together with other nonstructural proteins are responsible for the production of infectious viruses [65]. Moreover, Masaki and colleagues reported that NS5A physically interacts with core through its domain III and that effective production of progeny virions is directly linked to the levels of this interaction [66]. However, NS5A alone is the viral protein responsible for regulating the shift from HCV replication to virion assembly and the formation of viral capsids. It is also believed to be the viral switch between translation and replication [67]. To fulfill these unique roles, HCV NS5A recruits known, such as the membrane sorting protein Annexin A2, the apolipoprotein E, the double‐stranded RNA sensor PKR, and, as yet, unknown host factors that need to be regulated accordingly so that the virus life cycle can be completed [30, 33]. Thus, HCV NS5A modulates the expression of necessary host factors in a synergistic/additive way with HCV core, as it is often the case [39]. In contrast, crucial transcription factors that regulate multiple cellular events important for cell homeostasis and stimuli‐dependent gene expression, like c‐jun, AP‐1, and p53, have been shown to be differentially altered by HCV core and NS5A [68, 69, 70, 71, 72, 73]. This is true for other cellular processes, such as hepatic lipid accumulation, which is absolutely necessary for the completion of almost all stages of the viral life cycle [74] or the regulation of chemokine secretion [75]. In the light of these studies, the observed NS5A‐mediated down‐regulation of *HAMP* gene expression may be justifiable to the extent that NS5A is capable of fine‐tuning host gene expression to facilitate succession of viral life cycle steps. Overall however, HCV core accumulation was proven able to reinstate hepcidin expression by activating the appropriate signaling cascades, when needed, so that the net effect of HCV infection on hepcidin remains positive.

Finally, our results indicate that NS5A diminishes *HAMP* gene expression by acting as a zinc ion ‘sponge’, thereby causing imbalance in the MTF‐1/MRE/HAMP regulatory axis. Previous studies on the effect of JFH‐1 HCV on *HAMP* mRNA expression revealed a transient but significant reduction at the very early stages of infection (6‐ to 12‐h post‐infection) [28]. At that point, we attributed observation to a side effect of viral genome translation during the first round of virus replication. We now postulate that it may well be a result of NS5A production, at least in part.

In conclusion, we have shown that HCV NS5A protein is able to significantly diminish the expression of the iron‐regulatory protein hepcidin, leading to matching changes in other components of iron homeostasis. This reduction was caused irrespectively of major signaling events responsible for constitutive and HCV core‐mediated regulation of *HAMP* gene expression, like the BMP/SMAD pathway, but could be restored by HCV core when appropriate. A possible mechanism for the observed effect that involves a decrease in abundance of intracellular zinc ions and deregulation of the MTF‐1/MRE/hepcidin axis may be dependent on the avid production of NS5A during HCV RNA translation. Further repercussions of the down‐modulation NS5A confers on hepcidin and how these may be related to completion of specific parts of the viral life cycle and the well‐being of the host remain to be investigated.

## Conflict of interest

The authors declare that the research was conducted in the absence of any commercial or financial relationships that could be construed as a potential conflict of interest.

## Author contributions

UG, PF, AD, and AM conceived and designed the experiments. EKy, AD, PF, UG, SP, and EKa performed the experiments. AK, PT, and PE contributed with specific reagents, materials, and valuable suggestions. AD, PF, AM, and UG wrote and revised the manuscript. All authors have approved the manuscript for submission.

## Supporting information


**Fig. S1.** Western blot analysis of whole‐cell extracts from A1: Huh7 and HepG2 cells of figure 1A, A2: Huh7 cells of figure 1B, B1: Huh7 cells of figure 4A1, and B2: pTRE‐tight and NS5A cells of figure 4B2 against the HCV NS5A and core proteins. C: Western blot analysis of whole‐cell extracts from pTRE‐tight and NS5A cells against the STAT3 transcription factor. The expression of actin was monitored as internal control. Polypeptide molecular weights are given on the side in kDa. Individual gel photographs presented in this figure panel depict results from samples that were derived from the same experiment and processed in parallel.

## Data Availability

Data will be made available from the corresponding author upon reasonable request.
