# Supplementary figures and images for: The Hepatitis C virus NS5A and core proteins exert antagonistic effects on HAMP gene expression: the hidden interplay with the MTF‐1/MRE pathway
Source: FEBS Open Bio. 2020 Dec 13;11(1):237–50. doi: 10.1002/2211-5463.13048 (PMC7780115; doi:10.1002/2211-5463.13048)

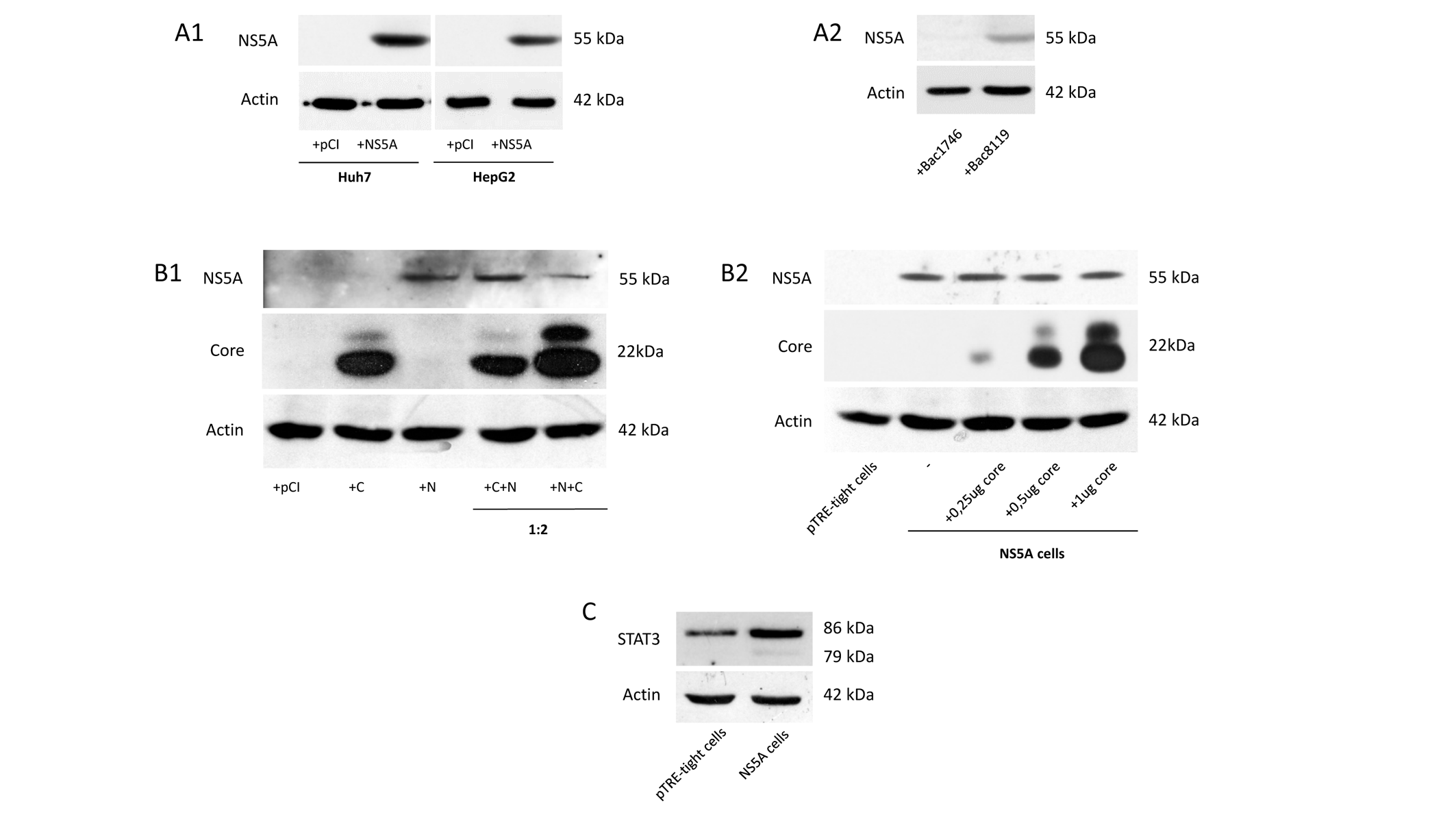

Supplement: Supplementary file 1 — Fig. S1. Western blot analysis of whole‐cell extracts from A1: Huh7 and HepG2 cells of figure 1A, A2: Huh7 cells of figure 1B, B1: Huh7 cells of figure 4A1, and B2: pTRE‐tight and NS5A cells of figure 4B2 against the HCV NS5A and core proteins. C: Western blot analysis of whole‐cell extracts from pTRE‐tight and NS5A cells against the STAT3 transcription factor. The expression of actin was monitored as internal control. Polypeptide molecular weights are given on the side in kDa. Individual gel photographs presented in this figure panel depict results from samples that were derived from the same experiment and processed in parallel. [file FEB4-11-237-s001.tif]
